# Neuronal Cytoskeleton in Intellectual Disability: From Systems Biology and Modeling to Therapeutic Opportunities

**DOI:** 10.3390/ijms22116167

**Published:** 2021-06-07

**Authors:** Carla Liaci, Mattia Camera, Giovanni Caslini, Simona Rando, Salvatore Contino, Valentino Romano, Giorgio R. Merlo

**Affiliations:** 1 Department of Molecular Biotechnology and Health Sciences, University of Torino, Via Nizza 52, 10126 Torino, Italy; carla.liaci@unito.it (C.L.); mattia.camera230@edu.unito.it (M.C.); giovanni.caslini@edu.unito.it (G.C.); simona.rando@edu.unito.it (S.R.); 2 Department of Engineering, University of Palermo, Viale delle Scienze Ed. 8, 90128 Palermo, Italy; salvatore.contino01@community.unipa.it; 3 Department of Biological, Chemical and Pharmaceutical Sciences and Technologies (STEBICEF), University of Palermo, Viale delle Scienze Ed. 16, 90128 Palermo, Italy; valentino.romano@unipa.it

**Keywords:** actin cytoskeleton, microtubules, GTPase signaling, small Rho GTPases, intellectual disability, neuronal networks, systems biology, Boolean modeling, protein:protein interaction network, pharmacological modulation

## Abstract

Intellectual disability (ID) is a pathological condition characterized by limited intellectual functioning and adaptive behaviors. It affects 1–3% of the worldwide population, and no pharmacological therapies are currently available. More than 1000 genes have been found mutated in ID patients pointing out that, despite the common phenotype, the genetic bases are highly heterogeneous and apparently unrelated. Bibliomic analysis reveals that ID genes converge onto a few biological modules, including cytoskeleton dynamics, whose regulation depends on Rho GTPases transduction. Genetic variants exert their effects at different levels in a hierarchical arrangement, starting from the molecular level and moving toward higher levels of organization, i.e., cell compartment and functions, circuits, cognition, and behavior. Thus, cytoskeleton alterations that have an impact on cell processes such as neuronal migration, neuritogenesis, and synaptic plasticity rebound on the overall establishment of an effective network and consequently on the cognitive phenotype. Systems biology (SB) approaches are more focused on the overall interconnected network rather than on individual genes, thus encouraging the design of therapies that aim to correct common dysregulated biological processes. This review summarizes current knowledge about cytoskeleton control in neurons and its relevance for the ID pathogenesis, exploiting in silico modeling and translating the implications of those findings into biomedical research.

## 1. Introduction

ID is a heterogeneous group of neurodevelopmental disorders (NDDs), usually diagnosed before the age of 18, characterized by significant limitations in both intellectual functioning (IQ < 70) and adaptive behavior as expressed in conceptual, social, and practical adaptive skills [1]. It affects 1–3% of the worldwide population, depending on the inclusion criteria, with a higher prevalence among males [2,3,4,5]. Because of its high frequency, limited treatability, and required lifelong care, ID has a dramatic social and economic impact.

ID is manifested as both syndromic and non-syndromic forms, depending on whether the disability is associated with other symptoms, and it is clinically classified referring to severity (mild, moderate, and severe) and penetrance (partially to fully penetrant) [1]. A phenotype-based cluster analysis was made by Kochinke et al., establishing gene–phenotype relationships and revealing compromised molecular processes that underlie specific ID subgroups. For example, genes involved in growth factor signaling pathways, such as the MAPK pathway, are enriched in comorbidities such as short stature and ectodermal anomalies as compared to all other ID-associated genes. Epilepsy, metabolic dysfunctions, and myopathy are instead co-occurring within a cluster of genes enriched with mitochondrial function. Microcephaly and behavioral abnormalities were linked to two clusters comprising genes enriched with chromatin-related functions.

ID mutations can be autosomal–recessive, autosomal–dominant (mostly de novo), or X-linked. The latter two are responsible for the higher prevalence of ID in males versus females.

The causes of ID are heterogeneous and still to be completely defined: it has been estimated that half of all cases are due to environmental factors such as intrauterine/neonatal insults (preterm-birth complications, intrapartum-related factors such as hypoxic-ischemic encephalopathy, and infections like meningitis and neonatal tetanus) and postnatal risk factors such as severe malnutrition during infancy. The other half of the cases are associated with genetic variants, highly heterogeneous, and only partially identified. According to the SysID database [1], 1454 ID genes (excluding 1224 annotated as low-confidence ID genes) have been identified, some of which code for proteins involved in the Rho GTPases signaling pathway, such as *OPHN1* (oligophrenin 1), *ARHGEF9* (Cdc42 guanine nucleotide exchange factor 9), *FGD1* (FYVE, RhoGEF, and PH domain-containing 1), *RAC1* (Rac family small GTPase 1), and *PAK3* (P21-activated kinase 3).

Based on a wealth of experimental data from animal models and cultured neurons, it is widely accepted that cognitive deficits in ID patients are linked to altered neuronal networking, impaired synaptic plasticity, and excitation/inhibition unbalance in the cerebral cortex and hippocampus, resulting in abnormal information processing [6,7,8,9,10,11].

## 2. From Genetics to Core Regulatory Modules

As genome-sequencing technologies improve and become accessible, more ID-causing mutations will surely be identified in patients. However, our mechanistic understanding of ID pathophysiology continues to lag behind the pace of gene discovery.

Considering the elevated number of risk genes and their heterogeneity, it is unlikely that each identified mutation represents an independent pathway that, when misregulated, causes a similar cognitive phenotype. On the contrary, it can be assumed that the identified mutations may converge to, or participate in, a limited number of core regulatory intracellular modules that are beginning to be identified, although they are not yet fully characterized. The dysfunction of different genes impacting the same process will result in analogous dysfunctions of the process itself. Thus, multiple genetic causes converge on a few common cellular outcomes and result in one overall phenotype. For this reason, an integrated approach that collects a large set of data but focuses on single biological processes is more suitable for furthering genetic diagnostics and developing treatment strategies to target shared pathways rather than single genes.

Three key questions arise: (i) What are the common core regulatory mechanisms dysregulated in ID? (ii) What are the key proteins (hubs; in gene network theory, hubs are defined as nodes with a high number of edges compared with other nodes) and/or posttranslational modifications at the basis of the cell endophenotype resulting in ID? (iii) Do we have adequate tools to identify and study such hubs and biological processes?

Integrative methods and data meta-analyses, protein::protein interaction (PPI) networks, and transcriptomics analysis coupled with gene ontology (GO) [12,13] have been successfully used to answer these questions, a general approach also known as SB. To reorganize the wealth of mutational data into biologically coherent modules, Kochinke et al. characterized the functional coherence and connectivity of a set of high-confidence ID genes using GO-based annotations and PPI databases. Eighty-six percent of these genes were found to be associated with at least one of 32 GO annotations, with the higher fold enrichment detected for transcription and chromatin regulation, metabolism, WNT, Hedgehog, MTOR, and MAPK signaling pathways, synaptic functioning, ubiquitination, cytoskeleton, and small GTPase signaling. Most ID proteins were also found to be co-expressed, especially in the hippocampus, and to physically interact with each other. Similarly, Liu et al. [14] organized 63 prioritized high-confidence ID genes based on biological annotations and PPI networks, showing that they tightly converge onto two cellular mechanisms: chromatin modification/transcriptional regulation and synaptic function. Moreover, co-expression networks revealed that the same genes are enriched in the cortex from the early fetal to late mid-fetal stages.

A second approach used RNA-seq data derived from the blood of patients harboring mutations in the ID genes *CCNT2* (cyclin T2), *CDK9* (cyclin-dependent kinase 9), and *TAF2* (TATA-box binding protein associated factor 2), all encoding for transcription factors [15]. Differentially expressed genes were functionally enriched in the GO classes cytoskeleton dynamics, GTPase activity, axonogenesis, synaptic plasticity, neuronal differentiation, and chromatin regulation.

A third approach adopted a systematic analysis by building a highly stringent PPI network from genes previously related to ID and global developmental delay (GDD) in the Human Phenotype Ontology database [16]. This analysis identified six genes defined as hubs and 166 brain-expressed proteins that have not been previously associated with ID and GDD. The six hubs included *CDC42* (cell division cycle 42) and *RAC1*, two known cytoskeleton regulators, *APP* (amyloid β precursor protein), involved in proliferation, cell–cell adhesion, migration, and synaptogenesis, *EP300* (E1A binding protein p300), important for genomic stability through chromatin regulation, *TP53* (tumor protein p53), and *GNB1* (G protein subunit β1).

**Figure 1:**
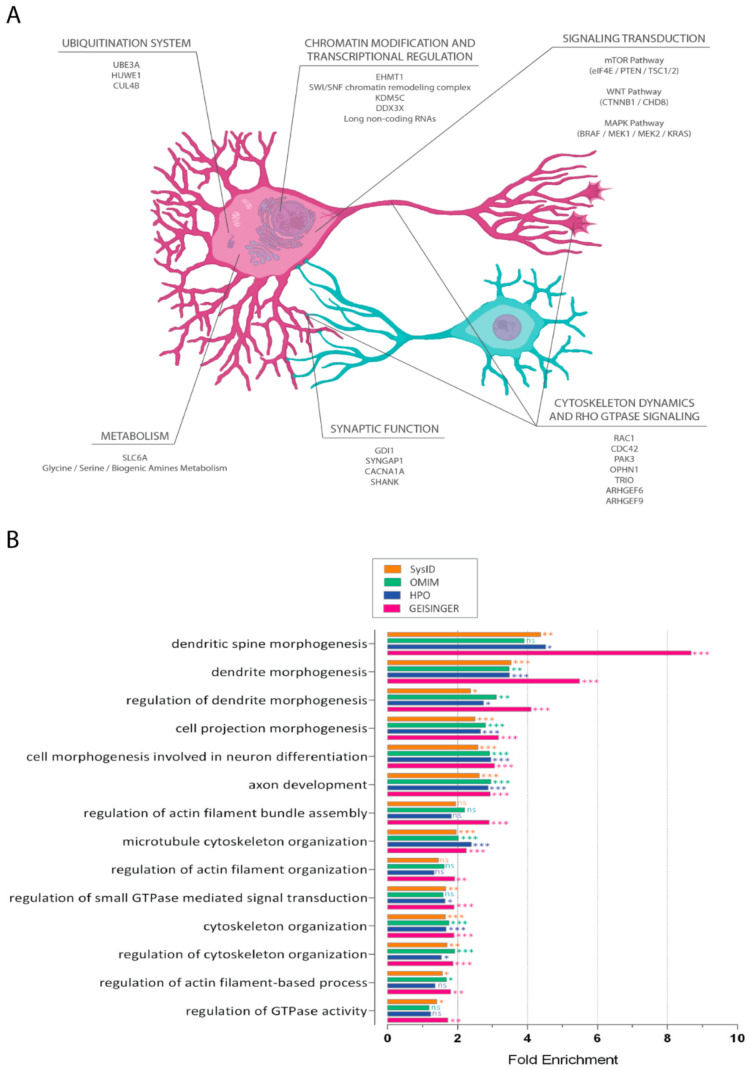
**Core regulations in intellectual disability.** (**A**). Deregulated biological processes identified in ID. The scheme illustrates the main intracellular processes identified via integrated analyses and their subcellular localization within hypothetical excitatory (in purple) and inhibitory (in blue) neurons. For each process, a few representative ID genes are reported. (**B**). GO terms [12,13] enrichment for ID-associated genes. The terms reported referring to biological processes linked to cytoskeleton activity and regulation. Four databases provided the list of ID-associated genes: HPO (Human Phenotype Ontology, in blue) [58], GEISINGER (in red) [59], OMIM (Online Mendelian Inheritance in Man, in green) [60], and SysID (in orange) [1]. *, **, and *** indicate *p* < 0.05, <0.01, and <0.001 respectively. n.s., not significant.

Overall, the SB approaches reported above identified the following wide biological processes on which ID genes converge (Figure 1A).

### 2.1. Chromatin Modification and Transcriptional Regulation

Many chromatin-modifying enzymes and other epigenetic regulators have been genetically associated with ID and other syndromes in which ID is one of the major clinical outcomes [17,18]. A catalog of 519 ID genes was enriched 2.7-fold in the GO terms chromatin binding, chromatin remodeling, and chromatin modification [1]. Mutations in *EHMT1* (euchromatic histone lysine methyltransferase 1) and SWI/SNF chromatin remodeling complex were shown to have a role in Kleefstra syndrome and Coffin–Siris syndrome [19,20], respectively; mutations in *KDM5C* (lysine demethylase 5C), encoding an eraser enzyme for di-methylated and tri-methylated histone H3 lysine 4, account for 2% of X-linked ID (XLID) [21]; mutations in *DDX3X* (DEAD-box helicase 3 X-linked), coding for an RNA helicase involved in post-transcriptional modifications, were found to be relevant in ID because of its fundamental role in neurite outgrowth and dendritic spine formation via modulation of *RAC1* transcription [22]. Interestingly, analysis of co-expression networks and genetic structural variants suggested a role for long non-coding RNAs in ID [23].

### 2.2. Signal Transduction

WNT, MTOR, and MAPK signaling pathways have been shown to play a central role in brain development. Perturbations of these pathways have been implicated in multiple neuropsychiatric disorders, including autism spectrum disorder (ASD) and ID [23,24,25]. Mutations in elements of the MTOR signaling pathway can affect the synaptic transmission and dendritic spine density, as shown by mutation of *EIF4E* (eukaryotic translation initiation factor 4E), a regulator of MTOR (mechanistic target of rapamycin kinase) translation [26], while loss-of-function (LOF) of upstream components of the MTOR pathway like *PTEN* (phosphatase and tensin homolog) and *TSC1/2* (TSC complex subunit 1 and 2, also known as tuberous sclerosis 1 and 2) results in overactive MTOR signaling, causing dendritic and axonal overgrowth, neuronal hypertrophy, and ASD-like behavioral patterns [27,28]. Similarly, mutations in WNT pathway components, like *CTNNB1* (catenin β1) and CHD8 (chromodomain helicase DNA binding protein 8), are associated with ID, as CTNNB1 is involved in synaptic function and its mutations are associated with deficits in intra-hemispheric connections, dendritic branching, long-term potentiation (LTP), and cognitive functions [29]. MAPK pathway dysregulation has been implicated in several syndromic ID forms. For instance, mutations in *BRAF* (B-Raf proto-oncogene, serine/threonine kinase), *MAP2K1* (mitogen-activated protein kinase kinase 1, alias *MEK1*), *MAP2K2* (alias *MEK2*), and *KRAS* (KRAS proto-oncogene, GTPase) are associated with cardio-facio-cutaneous syndrome, in which ID is present in the majority of patients [30]. The constitutively active KRAS^12V^ expressed in both forebrain excitatory and inhibitory neurons cause decreased excitatory transmission, accompanied by reduced hippocampal LTP [31].

### 2.3. Ubiquitination System

Mutations in several ubiquitination system genes have been linked to ID. Gain and LOF mutations in *UBE3A* (ubiquitin protein ligase E3A) have been associated with increased risk of ASD and ID through dysregulation of WNT and MTOR pathways [32,33]; the X-linked E3 ubiquitin ligase *HUWE1* (HECT, UBA, and WWE domain containing E3 ubiquitin protein ligase 1), which regulates both CTNNB1 and EIF4E, was associated to ID and ASD [34,35]; *CUL4B* (cullin 4B) LOF mutations [36] cause defects in dendritic spines, affecting their morphogenesis and plasticity in the hippocampus via the accumulation of its target TSC1/2, and through the subsequent overactivation of the MTOR pathway [37].

### 2.4. Metabolism

The contribution of metabolic dysregulations to ID is highly dependent on the period in which the defect becomes relevant (prenatal, early or late infancy, adolescence). For example, alterations in oxidative phosphorylation during the prenatal period lead to abnormalities in brain formation [38]. Creatine deficiencies, like the one caused by *SLC6A8* (solute carrier family 6 member 8, alias *SLC6A*) mutations, cause a mild to moderate ID phenotype [39]. A mutation prevalence study in 288 male patients presenting mild to severe XLID found that 2.1% of them carried a *SLC6A8* pathogenic mutation [40]. Disorders of glycine, serine, and biogenic amine metabolism may produce severe mental and motor disturbances, having a connection with the molecular process of synaptic function [38], as seen in the succinic semialdehyde dehydrogenase deficiency, which causes γ-hydroxybutyric aciduria and disorder of GABA metabolism [41]. Finally, the excess or unavailability of substrates (urea cycle disorders, organic acidurias) can cause a varying severity of the ID phenotype [42].

### 2.5. Synaptic Function

Synaptic signaling pathways, such as the Rab and Arf pathways, are commonly altered in ID. Genome-wide weighted co-expression network analysis showed specific enrichment for synaptic functioning [14]. Mutations in genes coding for the GDI1 (Rab GDP dissociation inhibitor 1) regulator are known to be involved in ID [43]. The lack of GDI1 impairs synaptic vesicles’ biogenesis and recycling in the hippocampus by defective endosomal-dependent recycling, leading to alterations in short-term plasticity [44]. Numerous genetic studies have shown that de novo missense variants of *CACNA1A* (calcium voltage-gated channel subunit α1 A), coding the α-1A subunit of the P/Q-type voltage-dependent calcium channel, cause congenital ataxia and ID [45,46]. De novo mutations of SYNGAP1 (synaptic Ras GTPase activating protein 1) are found in non-syndromic ID patients [47]. In 2019, more than 50 individuals with SYNGAP1-related forms of ID showing behavioral abnormalities, including generalized epilepsy and ASD, have been reported [48]. Notably, most mutations affecting synaptic functioning are linked with the cytoskeleton regulation, in particular that of actin, required for the optimal trafficking of neurotransmitter vesicles (presynaptically) and receptor turnover (postsynaptically) [49,50].

### 2.6. Cytoskeleton Dynamics and Rho GTPases Signaling

The altered control of cytoskeleton dynamics within developing neurons is a core dysfunction in ID as well as in Down syndrome (DS), Rett syndrome, Fragile X syndrome (FXS), and ASD. *CDC42* and WASP, known regulators of actin filament polymerization and branching, have been identified as hubs in PPI networks based on differentially expressed genes in DS [51]. Schizophrenia patients showed reduced actin polymerization in the brain, justifying the altered dendritic spine morphology and the reduced spine density [52].

Imbalance of actin cytoskeleton regulation has also been reported in FXS, a condition caused by an LOF mutation in FMR1 (FMRP translational regulator 1, also known as fragile mental retardation 1) [53]. Transcriptomic analysis on animal models of Rett syndrome revealed dysregulation of genes associated with cytoskeleton dynamics, actin polymerization, and focal adhesion [54]. Recent evidence suggests that dysfunction of Rho GTPases signaling contributes substantially to the pathogenesis of ASD: twenty genes encoding Rho GTPases regulators and effectors have been listed as ASD risk genes, representing 2.4% of the total [55]. Studies on the regulation of actin cytoskeleton dynamics in stem cells from ASD patients revealed altered dynamics of filament reconstruction upon activation of the Rho GTPases RAC1, CDC42, or RHOA (ras homolog family member A), showing shorter and less arborized neurites [56]. Expression and phosphorylation of cytoskeleton components were determined in the prefrontal cortex, hippocampus, and cerebellum of autistic-like C58/J mice. They revealed a region-dependent altered expression and phosphorylation patterns of Tau isoforms, associated with anomalous microtubule depolymerization and region-dependent changes in ADF/cofilin expression and phosphorylation associated, in turn, with abnormal actin filament depolymerizing dynamics [57].

Overall, strong evidence indicates that cytoskeleton dynamics isaffected by mutations detected in ID (Figure 1B) and other NDDs; in this review, we focus on cytoskeleton regulation in physiological conditions and its dysregulation in the ID context.

## 3. Cytoskeleton Functions in Neuronal Development

During development, neurons migrate to find synaptic partners and establish the complexity of the neuronal wiring. Neurite extension and navigation are possible thanks to the formation of the growth cone, a sensory-motile structure at the tip of the growing axon directed by chemotaxis [61]. The structure of the growth cone is characterized by a dynamic periphery, in which actin filaments extend and retract to explore the surrounding environment, and by a more stable center that forms the axonal shaft [62]. The interplay between microtubule assembly and actin dynamics is then essential for axonal elongation. The polarity of microtubules, essential for the directional transport of proteins and organelles [63], allows the sliding movement that supports axon formation, as tubulin monomers are continuously transported at the leading edge of the growth cone [64].

Microtubules’ and actin filaments’ polymerization result from the addition of α/β-tubulin and glomerular actin (G-actin), respectively [65,66]. The rate of filament elongation and morphogenesis depends on the concentration and availability of monomers but also on the presence of proteins that regulate the assembly/disassembly kinetics and those responsible for increasing the level of complexity for higher-order network structures [67,68]. While microtubules are the stiffest components of the cytoskeleton and can switch between a stably growing state and a rapidly shrinking one [69], actin filaments are less rigid and more organized, supporting the overall structure and allowing the motility of the leading edge [70].

It is assumed that growth cones are already provided with all the proteins necessary for synaptogenesis during their searching for contacts [71]. The protrusion of filopodia finger-like structures retract upon contact with the postsynaptic cell to form a vestigial presynaptic terminal. These filopodia are characterized by a less tight bundle that is more dynamic compared to the architectural stability of conventional filopodia [72]. This dynamism is required to let the nascent spine be perfectly aligned between the presynaptic active zone and the postsynaptic density (PSD). The juxtaposition is possible thanks to the presence of cell adhesion molecules that provide perfect docking geometries between the two membranes in the synaptic cleft [73]. The actin cytoskeleton is involved in spine morphogenesis, as it controls changes in spine shapes. Immature dendritic spines show linear and thin-like structures, but, after making contact with the presynaptic terminal, actin filaments begin to cluster and enlarge the contact surface to form a mature spine with a mushroom-like shape [74]. This is conceivable thanks to actin-related proteins that generate branched filaments, such as the ARP2/3 complex, balanced with capping proteins, such as CAPZ, to restrict their elongation and actin severing proteins, such as ADF/cofilin, that enhance filament disassembly [75]. In addition, several scaffold proteins contribute to the maturation of dendritic spines controlling actin dynamics, e.g., PSD95, SHANK, and SRCIN1 (also known as p140Cap) [76,77,78].

Synapses are not static formations. They undergo changes in postnatal life, while carrying out specific activities, e.g., learning, and in specific periods, e.g., synaptic pruning during adolescence. In particular, synaptic plasticity takes place in an activity-dependent manner: LTP is the result of strengthened synapses after high-frequency stimulation from the presynaptic terminal, while long-term depression (LTD) is a decrease in synaptic activity after low-frequency signals [79]. For LTP, the presence of NMDA-type glutamate receptors in the membrane of the postsynaptic cells allows the insertion of new AMPA receptors in response to high-frequency stimuli. The localization of these ionotropic, excitatory glutamate receptors leads to an increase in the postsynaptic current and consequently to a stronger connection in a positive feedback loop. Both AMPA receptors’ and NMDA receptors’ trafficking relies on the actin cytoskeleton [80,81,82,83].

### 3.1. The Core Regulation of Actin Dynamics

**Figure 2:**
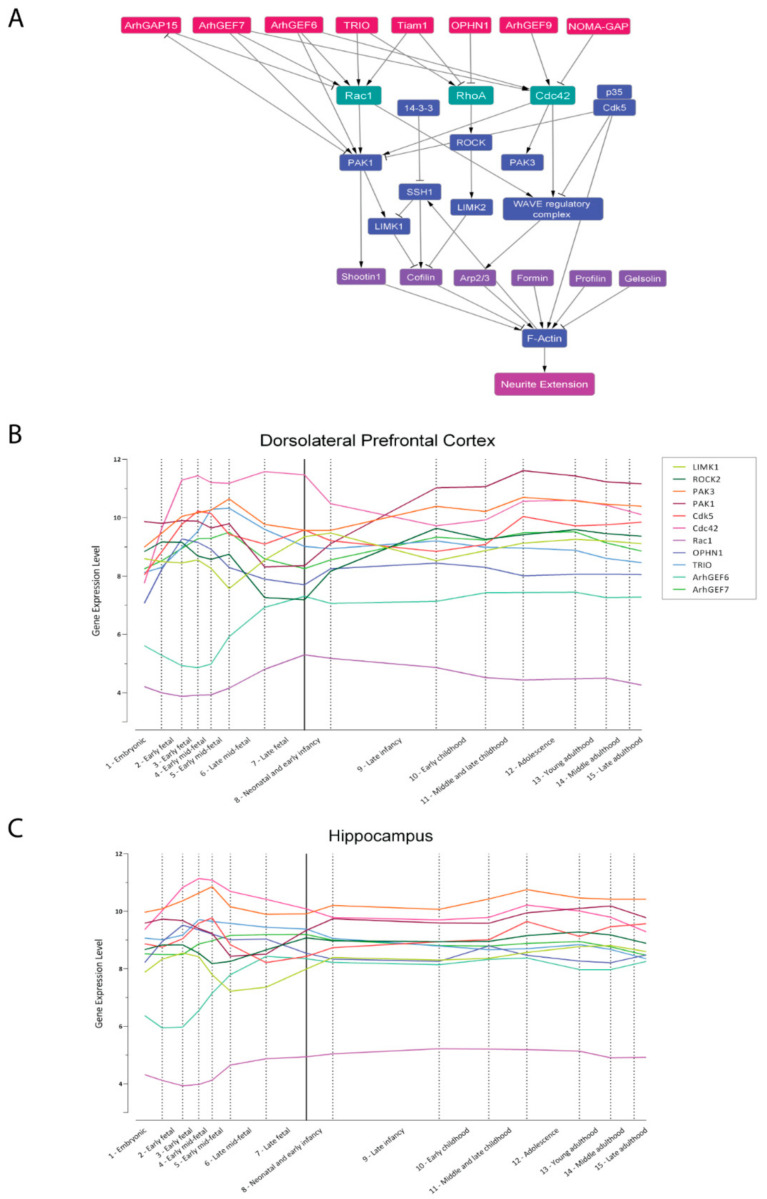
**Proteins involved in the regulation of neurite elongation.** (**A**). PPI network of the best-characterized components of the Rho GTPase signaling RAC1, RHOA, and CDC42 realized with Cytoscape [269]. Boxes represent the nodes (proteins), while the arrows indicate the edges (interactions). GTPases are reported in green, their GEFs and GAPs in red, their effectors in blue, and actin-binding or actin-modifying proteins in purple. Edges can be either “activatory” (arrowheads) or “inhibitory” (blunted lines). The “neurite elongation” node represents the phenotypic outcome. Acronyms are spelled out in the text. (**B**,**C**). Expression trajectories of ID-related genes in the human dorsolateral prefrontal cortex (**B**) and hippocampus (**C**). Quantile normalized gene-level expression values (log_2_ transformed) inferred from Human Brain Transcriptome database [270] were plotted against logarithmic age in days. The pattern was summarized by the smoothed curves of the expression values. Dashed lines divide periods of development and the solid line separates prenatal from postnatal periods. Individual genes are color-coded, legend in panel (**C**).

Alterations in neurites and spine morphology, as well as in neuronal migration properties, have been consistently associated with ID and other NDDs that include ID as a main and recurrent phenotype [84]. These developmental features rely on the proper actin cytoskeleton dynamics, as neurite outgrowth, axonal migration, synaptogenesis, and synaptic plasticity are the result of three main processes: fibrous-actin (F-actin) dynamics (elongation/severing/branching), actin–myosin contractility, and F-actin coupling with the extracellular matrix [85,86]. All three processes are regulated by a complex protein network in which the Rho-family small GTPases RAC1, CDC42, and RHOA emerge as hubs (Figure 2A).

This section illustrates in detail the key components of the signaling pathway responsible for the control of the dynamics of the actin cytoskeleton, focusing on the biochemical and cellular role of each protein and its links with neurological and cognitive deficits in human and animal models.

#### 3.1.1. Rho GTPases and Effectors

**RAC1:** RAC1 is a key regulator of neurite elongation, axon migration, synaptic function, and synaptic plasticity, as it promotes neurite outgrowth [85], spine formation and stabilization [87], and clustering of AMPA and NMDA receptors in the postsynaptic membrane [83,88], and it is essential for long-term synaptic plasticity in the hippocampus, which is the molecular mechanism at the base of learning and memory formation [89]. Formation and stabilization of integrin-dependent adhesion sites at membrane protrusion require local RAC1 activation followed by local RAC1 inactivation [90]. Moreover, the expression of constitutively active RAC1 inhibits NGF-induced neurite outgrowth [91], indicating that tight spatiotemporal regulation of RAC1 signaling is required for optimal neurite outgrowth.

Seven de novo missense *RAC1* variants have been reported in patients with mild to severe ID [92,93]. Among them, two function as dominant-negative alleles (p.Cys18Tyr and p.Asn39Ser), while one is a constitutively active allele (p.Tyr64Asp) [93]. For the other mutations, it is not clear if they generate dominant-negative alleles or if they could result in a condition of haploinsufficiency. Interestingly, the p.Cys18Tyr variant prevented GTP-mediated activation of RAC1 and prevented overexpression of the mutated RAC1 from inhibiting the induction of LTP in the hippocampus [94].

In mice, the deletion of *Rac1* in the ventricular zone of the telencephalon resulted in ventricles enlargement, impaired migration of median ganglionic eminence-derived interneurons, and impaired projection of commissural and corticothalamic axons. Interestingly, primary *Rac1*-deficient neurons had increased neurites formation and branching, indicating that RAC1 may be dispensable for neuritogenesis per se [95].

**CDC42:** CDC42 plays a critical role in neurite outgrowth [91], neuronal migration, and dendritic spines formation and maturation [96]. It is also essential for the establishment of neural polarity, as it promotes axon formation and elongation by regulating ADF/cofilin activity at the growth cone and by promoting microtubules stability through DPYSL2 (dihydropyrimidinase-like 2) [97,98].

Eight *CDC42* de novo missense mutations have been reported in 13 unrelated patients showing several developmental abnormalities, including ID and dysmorphic facial features [99,100,101,102]. In vitro studies and experiments involving *C. elegans* showed that these mutations result in proteins with altered activity and/or impaired target interactions, with some mutations acting as a gain of function and others acting as hypomorphs [101]. Notably, one missense mutation of *CDC42*, which has been described as a de novo mutation in one individual and inherited mutation in three related individuals, resulted in a hypomorphic allele associated with several developmental phenotypes, but not with ID [101]. Overall, the complex and heterogeneous set of developmental abnormalities associated with *CDC42* mutations may reflect different functional consequences of the single mutations.

Brain-specific *Cdc42*-KO mice die soon after birth and show a reduced cortical mass and a widespread loss of axonal tracts [97]. The effects of *CDC42* depletion in the postnatal brain have been assessed using *Cdc42^flox/flox^, Camk2a-CRE* mice, in which CRE recombinase is expressed in cortical pyramidal neurons and hippocampus starting from P16-P19. These mice showed reduced spine density and LTP in the hippocampus, together with memory deficits [103].

**RHOA:** Broadly speaking, the effects of RAC1 and CDC42 signaling on neurite outgrowth and dendritic spine formation are antagonized by RHOA signaling [85,90,104]. In particular, RHOA activity inhibits the formation of integrin-dependent adhesions [8], promotes neurite retraction by activating myosin 2a [105,106,107], and negatively regulates spine formation and maintenance [108].

Interestingly, *KCTD13* (potassium channel tetramerization domain containing 13) and *CUL3* (cullin 3), two genes linked to NDDs, are involved in RHOA ubiquitination [109,110,111], and RHOA inhibition rescues synaptic transmission, learning, and memory defects in *Kctd13*-KO mice [112,113]. These findings are consistent with the notion that RHOA dysregulation itself is linked to NDDs.

**PAK1:** Six PAK proteins have been identified in mammals. Based on their sequence homology, PAKs are classified into two groups, the first including PAK1, PAK2, and PAK3 and the second including PAK4, PAK5, and PAK6. All six PAKs are expressed in the nervous system with a different spatio-temporal pattern, with PAK1 and 3 being the most studied in the context of neuronal function [114]. Active RAC1 and CDC42 bind to the CRIB region of PAK1, relieving its autoinhibition and promoting its kinase activity [115,116,117].

PAK1 plays a critical role in both synaptic function and axon migration. *Pak1*-KO mice show impaired LTP at hippocampal CA1 synapses, reduced enrichment of F-actin at dendritic spines, and impaired NMDA-dependent ADF/cofilin phosphorylation [118]. Both overexpression and inhibition of PAK1 in the mouse developing brain led to profound defects in the migration of cortical neurons [119].

In humans, gain of function missense variants in *PAK1* have been associated with ID, macrocephaly, and seizures [120,121]. Interestingly, deficits in the PAK1 pathway may partially explain the impaired migration of GABAergic neurons in DS patients [122].

**PAK3:** Differently from PAK1, which is activated by both RAC1 and CDC42, PAK3 is mainly activated by CDC42 [123].

Mutations of *PAK3* are associated with XLID [124,125]. Two *PAK3* variants responsible for severe ID and corpus callosum agenesis (G424R and K389N) were shown to suppress kinase activity, increase the interaction between PAK3 and the guanine exchange factor ARHGEF6 (Rac/Cdc42 guanine nucleotide exchange factor 6, also known as α-PIX), and inhibit cell migration [124]. Another variant (R67C) inhibits the binding of PAK3 to CDC42, impairing PAK3 activation [123].

In mice, the latter variant impacts cognitive functions and adult hippocampal neurogenesis [126]. Likewise, *Pak3*-KO mice have no apparent defects in the actin cytoskeleton, but showed impaired hippocampal LTP, together with learning and memory deficits [127].

**LIMK1**: LIMK1 (LIM domain kinase 1) is a serine–threonine kinase that possesses two LIM domains, a PDZ domain, and a C-terminal kinase catalytic domain [128]. LIMK1 is a key downstream target of RAC1 signaling and is activated by PAK1 by phosphorylation at the Thr-508 residue [129]. Dominant-negative LIMK1 inhibits RAC1-stimulated lamellipodial protrusion [130], CDC42-induced filopodia formation, and RHOA-mediated stress fibers formation in Cos-7 cells [131].

Neurons of *Limk1*-KO mice showed reduced growth cone size and altered dendritic spine morphology [127].

In humans, heterozygous deletion of 27 genes, including *LIMK1*, results in Williams syndrome, a complex developmental disorder characterized by ID and impaired long-term memory [132]. Interestingly, *Limk1*^+/−^ mice also showed impaired long-term memory, together with reduced late-LTP in the hippocampus [133], indicating the LIMK1 haploinsufficiency in Williams syndrome patients may be causally related to memory defects. Unlike LIMK1, which is specifically expressed in the nervous system and enriched at mature synapses, LIMK2 is ubiquitously expressed [134,135], although it has been less studied. In the neuronal context, there is evidence for the role of LIMK2 in neurite outgrowth and neuronal migration [136,137].

**ROCK:** ROCK (Rho-associated coiled-coil containing protein kinase) is activated by active RHOA [138,139]. Two ROCK isoforms exist: ROCK1, which is prominently expressed in non-neuronal tissues such as liver and testis, and ROCK2, which is mainly expressed in brain and skeletal muscle [140]. ROCK activity stabilizes actin filaments by activating LIMK, which in turn inactivates ADF/cofilin [141,142]; on the other hand, ROCK promotes actomyosin contractility and stress fibers formation by phosphorylating MLC9 (myosin light chain 9) at Ser19, the same residue phosphorylated by MLCK (myosin light chain kinase) [143], and by phosphorylating MBS, the regulatory subunit of myosin light chain phosphatase [144,145]. A nonsense variant of *ROCK* was identified in an ID patient [146].

The pharmacological inhibition of actomyosin contractility inhibits actin retrograde flow and actin filaments’ severing, and promotes neurite outgrowth in the early stages of neuronal polarization [147,148], indicating that RHOA opposes neurite elongation by stimulating actomyosin contractility.

Notably, PAK1 inhibits MLCK [149], suggesting that RAC1 and RHOA act antagonistically on actomyosin contractility.

**Cdk5-p35:** CDK5 (cyclin-dependent kinase 5) is activated by binding with the specific protein partners CDK5R1 (cyclin-dependent kinase 5 regulatory subunit 1, also known as p35) and CDK5R2 (also known as p39) [150,151]. CDK5 is important for neuronal migration, neurite outgrowth, axon guidance, and synaptogenesis during brain development and for synaptic plasticity during adulthood [152,153]. CDK5 controls cytoskeleton remodeling by regulating Rho GTPases and by stabilizing actin filaments through p35-mediated-binding to F-actin [154]. CDK5 functions as a balance factor, as it can both facilitate RHOA-mediated growth cone collapse or dendritic spine retraction through phosphorylation of NGEF (neuronal guanine nucleotide exchange factor, also known as ARHGEF27) [155] or inhibit these processes by phosphorylating CDKN1B (cyclin-dependent kinase inhibitor 1B), and prevents RHOA activation by guanine exchange factors (GEFs) [156,157].

Similarly, CDK5 activates RAC1 via phosphorylation of KALRN (kalirin RhoGEF kinase) to promote dendritic spine stabilization [158] or inhibits RAC1 activation via phosphorylation of a RASGRF2 (Ras protein-specific guanine nucleotide releasing factor 2) or PPP1R9A (protein phosphatase 1 regulatory subunit 9A, also known as neurabin-I) [159,160]. CDK5 can also indirectly regulate CDC42-mediated dendrite outgrowth and extension via phosphorylation of NTR (neurotrophic receptor tyrosine kinase 2) [161].

In a mutation screening, novel silent mutations in *CDK5* and *p35* were identified: three intronic variations and four heterozygous variations in a cohort of 360 patients with non-syndromic ID, suggesting that these mutations and polymorphisms may contribute to ID phenotype [162].

#### 3.1.2. GAPs and GEFs

Small GTPases cycle between a GTP-bound active state and a GDP-bound inactive state. The most important regulators of small GTPases are GTPase-activating proteins (GAPs), which promote GTP hydrolysis, GEFs, which promote activation by inducing the release of GDP and the binding of GTP, and guanine dissociation inhibitors, which prevent GDP dissociation [163].

**OPHN1:** OPHN1 is an F-actin binding protein ubiquitously expressed in the central nervous system in both glial cells and neurons, where it mainly localizes at the tip of growing neurites, growth cones, and dendritic spines [164,165]. It shows GAP activity towards RHOA, and, to a lesser extent, towards RAC1 and CDC42 [164,166].

In humans, LOF mutations in *OPHN1* cause syndromic XLID, in which ID is associated with epilepsy, ventriculomegaly, and cerebellar hypoplasia [167,168,169].

*Ophn1*-KO mice recapitulate some aspects of the human phenotype, such as social, behavioral, and cognitive impairments, as well as ventricular enlargement and susceptibility to seizures [165,170]. At the cellular level, *Ophn1*-KO mice show hyperexcitability of the hippocampal network, associated with a reduced number of hippocampal GABAergic interneurons, impaired dendritic spine maturation, and short-term synaptic plasticity [165,170]. Moreover, *OPHN1*-deficient human iPSCs showed decreased neurogenic potential and impaired neurite elongation [171].

**ARHGAP15:** ARHGAP15 (Rho GTPase activating protein 15) is a RAC-specific GAP protein, expressed in both excitatory and inhibitory neurons of the adult hippocampus and cortex. It is a negative regulator of RAC1/RAC3 activity, and its loss results in the hyperactivation of the RAC1 pathway [172]. ARHGAP15 comprises a pleckstrin homology domain, which mediates its membrane localization and consequent activation via binding to the PI3K product phosphatidylinositol 3,4,5-trisphosphate [172].

*Arhgap15*-KO mice showed altered neuritogenesis and synaptic density, resulting in increased spike frequency and bursts, accompanied by poor synchronization. Its loss mainly impacts interneuron-dependent inhibition. Adult *Arhgap15*-KO mice showed defective hippocampus-dependent functions such as working and associative memories [172].

In humans, the loss of ARHGAP15 has been reported in a rare variant of Mowat–Wilson disease, which is characterized by severe neurological deficits, severe ID, speech impairment, and ASD [173,174].

**NOMA-GAP:** ARHGAP33 (Rho GTPase activating protein 33, also known as NOMA-GAP) is a multi-adaptor protein with GAP activity, and it is a major negative regulator of CDC42 [175]. NOMA-GAP has been shown to be essential for NGF-stimulated neuronal differentiation through the inhibition of CDC42 signaling and regulation of the ERK5-MAPK signaling [175]. *Noma-gap*-KO mice showed hyperactivity of CDC42 and reduced complexity of dendritic arborization [176].

**TRIO:** TRIO (trio Rho guanine nucleotide exchange factor) is a conserved Rho GTPase regulator that is highly expressed during brain development [177,178]. It contains two functional GEF domains: GEFD1, which regulates RAC1 and RHOG activity, and GEFD2, which regulates RHOA activity. It is involved in actin remodeling and it is necessary for cell migration and growth. TRIO controls, through RAC1 activation, cytokinesis, axon outgrowth, and guidance and modulates excitatory synaptic transmission [7,179]. In the developing hippocampal neurons, it limits dendrite formation without affecting the establishment of axon polarity. While *Trio*-KO has been shown to be embryonically lethal [180], hippocampus- and cortex-specific *Trio*-KO and heterozygous mice show progressive defects in learning ability, sociability, and motor coordination [178,181]. Whole-exome sequencing studies identified *TRIO* de novo mutations in several patients affected by NDDs in which ID appears as a prominent phenotype [182].

**ARHGEF6 and ARHGEF7:** ARHGEF6 and ARHGEF7 (Rho guanine nucleotide exchange factor 7, also known as βPIX) are GEFs of the Rho GTPases. ARHGEF6 has been shown to be specific for RAC1, activating and targeting it to membrane ruffles and focal adhesions [183]. On the other hand, *Arhgef6*-KO mice showed a significant reduction in the activity of both RAC1 and CDC42, but only at the hippocampal level [184].

Both proteins share an SH3 domain, a prerequisite for the binding with PAK1, PAK2, and PAK3 [185]. Santiago-Medina et al. [186] stressed the importance of the subtle regulation exerted on adhesion dynamics and membrane protrusions by PAK–ARHGEF6 and PAK–ARHGEF7 interactions during neurite outgrowth, as the partial inhibition of the interaction robustly stimulates neurite outgrowth and growth cone point contacts’ turnover, whereas the complete inhibition freezes it stabilizing adhesions.

Both ARHGEF6 and ARHGEF7 present the Dbl homology and pleckstrin domains, which possess RhoGEF activity. Moreover, an ARHGEF7 transcriptional isoform presents a PDZ target at the C-terminal, functional to the binding with PDZ protein, e.g., SHANKs (SH3 and multiple ankyrin repeat domains), at the excitatory synaptic sites [187].

Mutation screening of 119 patients with nonspecific ID revealed a T > C variant in the first intron of *ARHGEF6* (c.166-11T > C) [188,189], although the pathogenicity of this specific variant was then questioned [190]. In addition, a male patient with severe ID, carrying a molecularly unbalanced translocation (X;21) disrupting *ARHGEF6*, was then identified [188].

*Arhgef6*-KO mice showed an increased dendritic length of hippocampal pyramidal neurons, reduced spine synapses, an overall reduction in early-phase LTP, and an increase in LTD, together with impaired spatial and complex learning and less behavioral control in mildly stressful situations, resembling the human ID phenotype, thus validating *Arhgef6*-KO mice as a proper ID animal model [184].

For what concerns ARHGEF7, the case of two siblings presenting generalized epilepsy and ID was reported, consequently to the 13q34 deletion. This genomic locus contains two protein-coding genes, SOX1 (SRY-box transcription factor 1) and ARHGEF7, thereby supporting the possible contribution of ARHGEF7 haploinsufficiency to the pathogenic phenotype [191].

*Arhgef7*-KO mice showed embryonic lethality at E9.5; for this reason, the in vivo role of ARHGEF7 was investigated through heterozygous or cortex-specific KO mice [192,193]. These models demonstrated that ARHGEF7 is essential in both neuritogenesis and synaptogenesis during cortical and hippocampal development, since its loss results in extensive loss of axons and reduced dendritic complexity, as well as in a decrease of synaptic density. Furthermore, *Arhgef7* heterozygous mice exhibited impaired social interactions [192].

**ARHGEF9:** ARHGEF9 is a brain-specific GEF that specifically activates CDC42 [194]. It regulates, through the recruitment and activation of CDC42, the clustering of GPHN (gephyrin) at postsynaptic sites [195]. GPHN clusters, in turn, promote postsynaptic clustering of glycine receptors and GABA_A_ receptors [196].

*Arhgef9*-KO mice showed reduced GABA_A_ receptor clusters at dendritic spines, enhanced LTP, increased levels of anxiety, and impaired spatial learning [197].

In humans, *ARHGEF9* mutations cause an XLID syndrome associated with seizures and facial dysmorphism [198,199,200].

**TIAM1:** TIAM1 (TIAM Rac1 associated GEF 1) is a GEF protein highly expressed in the developing nervous system that activates RAC1 and, to a lesser extent, CDC42 and RHOA [201,202]. RAC1 activation by TIAM1 is required for neurite outgrowth induced by NGF/NTRK1, BDNG/NTRK2, and Ephrin/Eph signaling [203,204]. Moreover, suppression of TIAM1 activity leads to defects in axonogenesis and radial migration [205,206]. TIAM1 is also required for spine formation and morphogenesis in response to various extracellular signals. In particular, Eprin-B1/EphB2 signaling promotes spine development by activating RAC1 through TIAM1, while NMDA-mediated calcium influx at glutamatergic synapses activates a CAMK2 (calcium/calmodulin-dependent protein kinase II)-TIAM1 complex that persistently activates RAC1, leading to LTP and spine enlargement [207,208]; interestingly, knock-in mice harboring a mutation that inhibits the formation of the CAMK2–TIAM1 complex showed reduced RAC1 activity and memory deficits [209].

TIAM1 activity seems to be exquisitely relevant for granule neurons of the dentate gyrus, as TIAM1 knock-down (KD) in these cells led to a decreased number of glutamatergic synapses expressing AMPA receptor and to an increased spine length, while no effect was observed upon TIAM1 KD in CA1 neurons [210].

Consistent with this finding, *Tiam1*-KO mice showed defective maintenance of the dendritic arborization and impaired stabilization of dendritic spines in the granule neurons [211]. Strikingly, these mice showed enhanced contextual learning and memory [211]. For this reason, and considering that TIAM1 is overexpressed in DS patients, the authors of this study speculated that elevated levels of TIAM1 contribute to the learning and memory deficits associated with DS.

#### 3.1.3. Actin Binding Proteins

The effects of RAC1, CDC42, and RHOA on actin dynamics are mediated by actin-binding proteins (ABPs), which are classified according to their activity: actin depolymerization, such as ADF/cofilin, branching, such as ARP2/3, severing, such as GSN (gelsolin), bundling, such as fascin family proteins, and nucleotide exchanging, such as profilin family proteins [68].

**ADF/cofilin:** LIMK1 inhibits ADF/cofilin proteins by phosphorylation at the Ser3 residue [212,213].

This protein family is composed of three isoforms: DSTN (destrin, actin depolymerizing factor, also known as ADF), CFL1 (cofilin 1), which is the most expressed in the central nervous system, and CFL2, which is specifically expressed in muscle tissue [214,215]. Since most studies addressing the roles of ADF/cofilin do not specify the isoform and most antibodies do not differentiate between these isoforms, this protein family is referred to simply as ADF/cofilin.

ADF/cofilin binds to ADP-actin, increasing the depolymerization rate of the pointed end and causing the severing of actin filaments [131,216]. This leads to an increase in G-actin availability and the number of barbed ends, resulting, at physiological ATP-actin concentrations, in actin reorganization and promoting axon elongation [97,217,218]. Importantly, not only the balance but also the cycling between the active and inactive forms of ADF/cofilin plays a role in modulating actin dynamics [97,219,220].

**SSH:** Proteins of the Slingshot family (SSH1-3 in mammals) dephosphorylate ADF/cofilin at Ser3 [221], thereby controlling actin dynamics and reorganization. SSH proteins mediate NGF-induced neurite extension. SSH1 and SSH2 KD suppress neurite extension by increasing the concentration of the non-phosphorylated form of ADF/cofilin [222].

**YWHAZ:** YWHAZ (tyrosine 3-monooxygenase/tryptophan 5-monooxygenase activation protein zeta, also known as 14-3-3 ζ) is an adaptor protein that affects actin dynamics via the stabilization of phospho-ADF/cofilin [223] and the regulation of SSH and LIMK1 [224]. Additionally, it has been shown that 14-3-3 ζ inhibits the ubiquitin-mediated degradation of δ-catenin [225], a component of the cadherin–catenin cell adhesion complex, which in turn inhibits RHOA and activates CDC42 and RAC1 [226,227,228].

14-3-3 *ζ*-KO mice present reduced spine density, stressing the importance of this protein in the regulation of the actin cytoskeleton [229].

**ARP2/3:** The ARP2/3 complex is a heptameric complex formed by ACTR2 (actin-related protein 2, also known as ARP2), ACTR3 (also known as ARP3), and ARPC1-5 (actin-related protein complex 1–5) [230]. It binds existing actin filaments and initiates the formation of new filaments that branch off the existing filaments at an angle of about 70° [231]. The axon guidance molecules VEGF and SEMA3A affect actin dynamics at the growth cone by increasing and decreasing ARP2/3 activity, respectively [232,233]. Thus, ARP2/3 is essential for neuronal migration [74] but also for spine formation, maturation, and maintenance [234,235].

The postnatal loss of ARPC3 in forebrain excitatory neurons leads to progressive spine loss and defective LTP-induced spine volume expansion [235]. Moreover, ARP2/3 activity is required for the maturation of filopodia into spines and for the recruitment at the postsynaptic membrane of AMPA receptors, a process that is essential for the functional maturation of excitatory synapses [236]. The activity of ARP2/3 is also controlled by PAK1, which can phosphorylate ARPC1 promoting F-actin polymerization and branching [237].

**NPFs:** The activity of nucleation-promoting factors (NPFs) is required to activate the nucleation and branching activity of the ARP2/3 complex. These factors include WASP (Wiskott–Aldrich syndrome protein), N-WASP (neural WASP), the WAVE regulatory complex (WRC) formed by WASF1 (WASP family member 1), CYFIP1 (cytoplasmic FMR1 interacting protein 1), ABI2 (abl interactor 2), NCKAP1 (NCK associated protein 1), and BRIK (BRICK1 subunit of SCAR/WAVE actin nucleating complex), or paralogues of these, and the WASH complex, formed by WASHC1-5 (WASH complex subunit 1–5) [230,238]. The activity of NPFs is controlled by Rho GTPases; in particular, active RAC1 and CDC42 activate N-WASP and WASP, respectively, by binding to their CRIB region [239,240]. Active RAC1 has also been shown to activate WRC [241]. Notably, dominant-negative WASF1 abolishes the formation of RAC1-dependent lamellipodia and RAC1-dependent neurite extension [242].

Strong genetic evidence indicates that alterations in the NPFs-ARP2/3 signaling module may lead to ID: copy number variants of the chromosomal region 15q11-q13, encompassing *CYFIP1*, were identified in patients with ASD and ID [243], with several studies indicating a pathogenic role for both increased and decreased CYPFI1 dosage [244,245,246]; 21 de novo missense *CYPFI2* variants, most of which were shown to impact on WRC-mediated actin remodeling, have been reported in 37 ID patients [247,248]; WASHC4 has been identified as an autosomal recessive ID gene [249,250]; *NCKAP1* variants predicted to be deleterious for protein function have been associated with ID [251]; *ABI2* is a candidate autosomal recessive ID gene [252]; de novo splice site mutations of *WASHC5* were shown to cause Ritscher–Schinzel/3C syndrome, a disorder characterized by several phenotypes, among which ID [253].

**GSN:** GSN (gelsolin) acts by severing actin filaments and capping free barbed ends [254]. Its depletion in hippocampal neurons increases the number of filopodia by reducing their retraction [255]. GSN is recruited to dendritic spines following LTD [256], presumably by the increase in calcium concentration [257], suggesting its involvement in synaptic plasticity.

**FMN2:** FMN2 (formin 2) is an ABP that belongs to the family of formin homology (FH) domain proteins. Since it is involved in the maturation of tip adhesion, it is essential for the generation of traction forces by filopodia and the stabilization of the growth cone [258]. By binding to the actin cytoskeleton, it functions as a clutch with the extracellular matrix at adhesion sites. FMN2 was found localized to ventral actin stress fibers in fibroblasts [258] and *punctae* along dendrites in neurons [259].

Notably, *FMN2* truncating mutations in two consanguineous families lead to decreased spine density and non-syndromic autosomal-recessive ID [259].

**Profilins:** Profilin family proteins (PFN1-4) promote the conversion of ADP-actin into ATP-actin, thus providing the actin monomers necessary to sustain barbed end elongation [260]. In striking contrast, low levels of profilin can also inhibit actin polymerization by sequestering actin monomers [261,262,263]. Profilins may have a role in the stabilization of spine morphology [264], and it is involved in the regulation of actin polymerization in growing neurites, as both overexpression and expression of dominant-negative profilin lead to impaired neurite outgrowth [265].

**SHTN1:** PAK1 phosphorylates SHTN1 (shootin 1), promoting its interaction with F-actin [266]. SHTN1 physically interacts with L1-CAM and F-actin, thus allowing the force generated by actin retrograde flow to be transmitted to the extracellular matrix and coupling actin polymerization with neurite elongation [267,268].

Interestingly, SHTN1 mRNA was found to be consistently down-regulated in blood samples of ID patients harboring mutations in the transcription factors CCNT2, CDK9, and TAF2 [15].

### 3.2. Synaptogenesis and Synaptic Plasticity

A set of mutations identified in ID specifically affect spine development and morphological changes during maturation. In this section, we summarize the most relevant findings.

**KALRN:** KALRN (kalirin) is a GEF for RAC1 [271]. In mice, KALRN expression increases at two weeks of age, a key time for synaptogenesis [272]. *Kalrn*-KO mice showed decreased synaptic density in the apical dendrites of CA1 hippocampal neurons, along with learning deficits [273].

**PPP1R9A and PPP1R9B:** PPP1R9A (neurabin -I) and PPP1R9B (also called neurabin-II or spinophilin) show F-actin cross-linking and phosphatase activity. They are enriched in dendritic spines [274] and are likely to influence dendritic spine morphology and function through their interaction with F-actin [275].

*Ppp1r9b*-KO mice have a reduced number of filopodia and an increase in spine density [276].

**ARHGEF2:** Neurabin-I and Neurabin-II interact with the Rho GEF ARHGEF2 (also known as Lfc). This interaction selectively regulates Rho-dependent organization of F-actin in spines; ARHGEF2 is maintained inactive/sequestered through the interaction with microtubules and targeted to dendritic spines as a result of the interaction with Neurabin-I and Neurabin-II [277].

*ARHGEF2* mutation leads to reduced activity of the RHOA pathway. A homozygous frameshift mutation in the *ARHGEF2* is associated with ID, midbrain–hindbrain malformation, and mild microcephaly in a consanguineous family of Kurdish–Turkish descent [278].

**SHANK:** SHANK family proteins are higher-order organizing scaffold proteins of the PSD. They are known to interact with the ABP DBNL (drebrin like) in the PSD to promote the reorganization of actin after stimulation [279]. SHANK proteins activate RAC1 signaling at the PSD by recruiting ARHGEF7 through its PDZ C-terminal domain [187]. Indeed, heterozygous mice lacking a SHANK3 C-terminus have an impaired actin polymerization and a consequent decrease of NMDA receptor delivery to the postsynaptic plasma membrane [280]. SHANK3 can also directly interact with the ARP2/3 complex to increase F-actin level by decapping the barbed ends of actin filaments, thus promoting filament extension [281]. A large variety of alterations in SHANK proteins are grouped as “shankopathies” and are linked with NDDs characterized by alterations in the actin cytoskeleton network [282].

It has been proposed that in the 22q13 deletion syndrome, an NDD characterized by ID, the disruption of *SHANK3* is responsible for the clinical disorder [283,284,285].

**CAMK2:** The activity of CAMK2, which is stimulated upon the increase in intracellular calcium concentration, is essential for AMPA receptor delivery to the membrane of silent synapses and SYNGAP activation [48,286]. Whole-exome sequencing identified 19 rare de novo variants of CAMK2A and CAMK2B in 24 unrelated ID patients [287].

### 3.3. The Role of Microtubules in ID

Microtubules are basic elements of the cytoskeleton and actively participate in most neurodevelopmental processes. Neurons depend on microtubule dynamics for cell division, axon guidance, intracellular trafficking, and synapse formation [288]. They are constituted by heterodimers of α- and β-tubulin that associate to form a hollow cylinder. The stable to dynamic microtubules ratio is significantly higher in the neurite that is meant to form the axon as compared to the other neurites, indicating that stable microtubules are essential for axon specification [289,290]. Microtubules’ polarity is required to deliver various cargoes to the correct location within the cell to assure axonal trafficking and to maintain the correct neuronal morphology. Microtubules also play a crucial role in spinogenesis, as dynamic microtubules penetrate dendritic spines to modulate their morphology by interacting with a large variety of microtubule-associated proteins [291,292,293]. Some of them act directly on microtubules to affect their assembly or stability, while others act indirectly by modulating tubulin level or intracellular transport; for example, severing proteins are essential to increase tubulin monomers’ availability and to reorganize microtubules’ scaffold architecture [294], while microtubule plus-end tracking proteins (+TIPs) are responsible for the regulation of microtubules by interacting with the plus ends and by functioning as a scaffold for other regulatory proteins [295,296]. Cytoplasmic linker proteins (CLIPs), a subgroup of +TIPs, are fundamental for microtubule invasion into the growth cone leading edge and into nascent dendritic spines [297,298].

Many microtubule-associated genes are linked to ID and to other NDDs in which ID appears as a prominent and recurrent phenotype: *ADNP* mutations are associated with ASD; *ASPM, MCPH1, STIL, CDK5RAP2, CENPJ, PRUNE1,* and *KIF20* mutations are associated with microcephaly; *TUBB2B* mutations are associated with polymicrogyria; *LIS1*, *DCX,* and *TUBA1A* are linked to lissencephaly [288].

**KIF1A, KIF4A, KIF5C, and KIF7:** Kinesins are motor proteins that move along microtubules in an anterograde fashion, transporting their cargo towards microtubules’ plus end. KIF1A (kinesin family member 1A) is selectively expressed in neurons, and its partial or total depletion results in the disruption of axonal and dendritic transports [299]. A dominant de novo missense mutation in *KIF1A* was found in a patient with non-syndromic ID [300], and other de novo mutations were found in six patients affected by severe early-infantile neurodegenerative syndrome [301]. Next-generation sequencing revealed mutations in other kinesin family members such as *KIF4A* and *KIF5C* for which the causative role in ID is supported by evidence obtained using KO models [302]. Mutations in *KIF2A* and *KIF5C* were reported in patients with malformations of cortical development presenting ID [303], and homozygous mutations in *KIF7* were found in patients with ciliary disorders in which ID is part of the phenotype [304].

**KIFBP:** KIFBP (kinesin family binding protein) is a modulator of kinesins activity. Homozygous nonsense mutations of KIFBP are associated with Goldberg–Shprintzen syndrome, which is a form of syndromic ID [305].

**CHAMP1:** CHAMP1 (chromosome alignment maintaining phosphoprotein 1) is a zinc finger protein that regulates chromosome segregation during mitosis. De novo *CHAMP1* mutations are associated with GDD and ID [306,307,308].

**CLIP1:** CLIP1 (CAP-Gly Domain Containing Linker Protein 1) is a +TIP that regulates microtubule growth and bundling. Next-generation sequencing revealed an autosomal recessive form of ID associated with a nonsense variant in *CLIP1* in an Iranian consanguineous family [309].

**KATNAL1:** KATNAL1 (katanin catalytic subunit A1 like 1) is one of the two major catalytic subunits of the microtubule-severing enzyme Katanin, together with KATNAL2. Three unrelated patients with heterozygous de novo deletions encompassing 13q12.3 presented moderate ID phenotype; since this region contains *KATNAL1*, this gene has been proposed as a candidate ID gene [310].

**MID1 and MID2:** MID1 (midline 1) and MID2 are E3 ubiquitin ligases that have a role in microtubule stability and organization. *MID1* mutations are associated with Opitz G/BBB syndrome, in which mild to severe ID can appear [311]. A *MID2* missense mutation that disrupts its interaction with microtubules is associated with XLID [312].

**CDKL5:** CDKL5 (cyclin-dependent kinase-like 5) is a kinase protein essential for brain development. CDKL5 interacts with IQGAP1 (IQ motif containing GTPase activating protein 1), through which it forms a functional complex with its effectors RAC1 and CLIP1, MAPRE2 (microtubule-associated protein RP/EB family member 2), MAP1S (microtubule-associated protein 1S), ARHGEF2, and SHTN1 [313,314,315], thus stressing its regulation over cytoskeleton dynamics, in particular over microtubules. *CDKL5* mutations cause the so-called CDKL5 deficiency disorder in which severe ID is one of the most important clinical manifestations [316].

## 4. Cytoskeleton in Non-Neuronal Cells and ID

**Figure 3:**
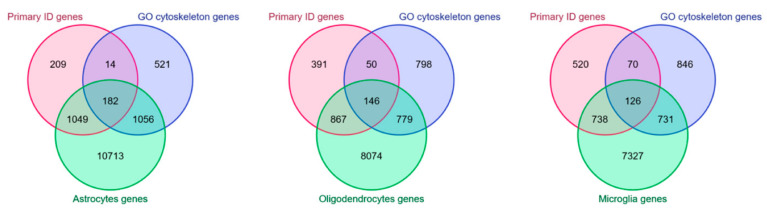
**Cytoskeleton-related ID gene expression in non-neuronal cells.** Venn diagrams created with the multiple list comparator tool by molbiotools [345] showing the intersections between the lists of primary ID genes reported in the SysID database (red) [1], genes associated to the GO terms [12,13] “regulation of GTPase activity” and “cytoskeleton organization” (blue), and genes expressed in astrocytes (on the left), oligodendrocytes (at the center), and microglia (on the right) (green). The list of genes expressed in non-neuronal cell types was obtained from an RNA-seq dataset [346].

Astrocytes, oligodendrocytes, and microglia cells express a fair fraction of ID genes, including those involved in Rho GTPases signaling and cytoskeleton organization (Figure 3). For this reason, and considering that these cells contribute to cognitive functions, it is reasonable to hypothesize that mutations in these genes might lead to ID not only by affecting neuron functions but also in a non-neuron autonomous manner. In the following section, we will review some of the literature on this subject.

**Astrocytes:** It is well established that astrocytes play a role in learning and memory formation [317,318]. For example, inhibition of lactate release by astrocytes impairs long-term memory formation [319], while chemogenetic or optogenetic activation of astrocytes in the CA1 region of the hippocampus during learning enhances memory formation [320].

The actin cytoskeleton is involved in many aspects of astrocyte physiology and function, such as the plasticity of the perisynaptic astrocytic processes [321], small membrane protrusions that contact synapses and regulate synaptic transmission [322], glial scar formation [323,324], and vesicle trafficking [325].

Therefore, one might speculate that defects in cytoskeleton dynamics may lead to ID by affecting astrocyte functions. In line with this hypothesis, astrocytes of *Ophn1*-KO mice are less ramified and show altered migration and glial scar formation [326]. Moreover, mice deficient for ARNTL (aryl hydrocarbon receptor nuclear translocator like, also known as Bmal1), an essential component of the molecular clockwork driving circadian rhythms, showed severe cognitive deficits, associated with a reduction of perisynaptic astrocytic processes covering hippocampal mossy fiber synapses, which is probably due to a reduction of RHOA activity in *Arntl*-deficient astrocytes [327].

The generation of astrocyte-specific *Ophn1*-KO and *Arntl*-KO mice may elucidate the non-neuron autonomous contribution of these proteins.

**Oligodendrocytes:** Oligodendrocytes, the myelin-forming cells of the central nervous system, are essential to establish neuronal networks with proper functions [328,329]. Myelination of newborn synapses is a key process during learning and memory formation, indicating a role for oligodendrocytes in cognitive functions [330,331]. The actin cytoskeleton participates in myelination in two steps: first, actin filaments’ assembly drives oligodendrocytes’ branching, and second, their depolymerization induces myelin wrapping [332]. This regulation underlies the importance of actin dynamics in oligodendrocytes, with actin-depolymerizing proteins like GSN and ADF/cofilin being among the most abundant transcripts in oligodendrocytes [333,334]. Myelin defects have been reported in DS patients [335,336] and non-syndromic XLID patients with mutations in *PAK3* [337,338]. PAK3 is highly expressed in oligodendrocyte progenitor cells, and it is essential for their differentiation into mature oligodendrocytes [339,340,341]. Notably, P14 (but not P60) *Pak3*-KO mice showed myelination defects of the axons of corpus callosum [341], a phenotype that is reminiscent of corpus callosum agenesis observed in ID patients harboring *PAK3* mutations.

**Microglia:** Microglia cells, the resident innate immune cells of the brain, are involved in the regulation of brain development by promoting both synaptic pruning and synapse formation [342]. Interestingly, mice with autophagy-deficient microglia showed impaired synaptic pruning and, consequently, altered brain connectivity, leading to social and behavioral defects [343]. Since microglia engulf synapses during pruning [342,344] and considering that engulfment requires cytoskeleton rearrangements, it would be interesting to investigate the role of cytoskeleton dynamics in synaptic pruning.

## 5. In Silico Modeling of Cytoskeleton Regulation

SB approaches complement the wealth of experimental and mutational data derived from small-scale studies and high-throughput methods with computational and mathematical modeling to reconstruct the dynamic organization of a specific cellular regulatory network, a task that is often out of reach of molecular technologies. Moreover, computational models can be implemented with existing data to raise predictions of how the biological system would behave in particular conditions. Our ability to model a complex regulation of connected elements in silico has largely improved, allowing us to:

‑ simultaneously consider a large number of interacting proteins and link their relationships with emerging phenotypes
‑ elaborate hypotheses and design new experiments
‑ search for biomarkers and druggable targets for translational purposes

SB studies are performed with bottom-up (dynamic models such as Boolean models) or top-down (statistical analyses and static networks) procedures. A Boolean model was implemented to study the molecular dynamics underlying the behavior of the neuronal growth cone during axon growth and guidance. Interestingly, simulations performed with mutant networks suggest that many mutations underlying ID and ASD affect the motility of the growth cone and in particular the formation of filopodia and lamellipodia [347]. To our knowledge, no studies have currently applied this type of modeling to study the regulatory module underlying neurite elongation.

Bottom-up pipelines start with a survey and manual curation of the scientific literature and databases looking for key genes, proteins, and interactions regulating the process under investigation, e.g., neurite extension, in order to derive a graphical depiction of the network in which nodes are genes/proteins and edges are the interactions among them. Here, we provide an example of a protein regulatory network for neurite elongation centered on GTPases (Figure 2A). The graphical map can be translated into a computational and/or mathematical model for dynamic simulations of the biochemical network in different contexts.

There are different frameworks for mathematical/computational modeling (ODEs, Boolean, agent-based), which differ in their trade-off in terms of biological complexity, scalability, or simulation reliability. Referring to Boolean models, there are only two mutually exclusive states for each node, the logic 1 and 0. The initial graphical map is rewritten converting the interactions between proteins/genes to Boolean functions by using the three logical operators: AND, OR, and NOT. As an example, in Table 1**,** we show the Boolean model corresponding to the network of Figure 2A rewritten with the >syntax of the ‘BoolNet’ R package [348] and simplified by removing nodes that lack downstream targets in our reconstruction of the network (i.e., PAK3) or that exert a redundant effect with other nodes (i.e., Gelsolin and ArhGEF9), thus reducing the computational cost.

Simulations can be run by choosing the initial conditions to be used (exhaustive, chosen, random), i.e., selecting which node is set as active (1) or inactive (0). During the simulation, the nodes state can be either synchronous or asynchronous: in the synchronous type, the state is updated simultaneously, while in the asynchronous type, only one transition function—randomly chosen—is updated at each simulation.

**Figure 4:**
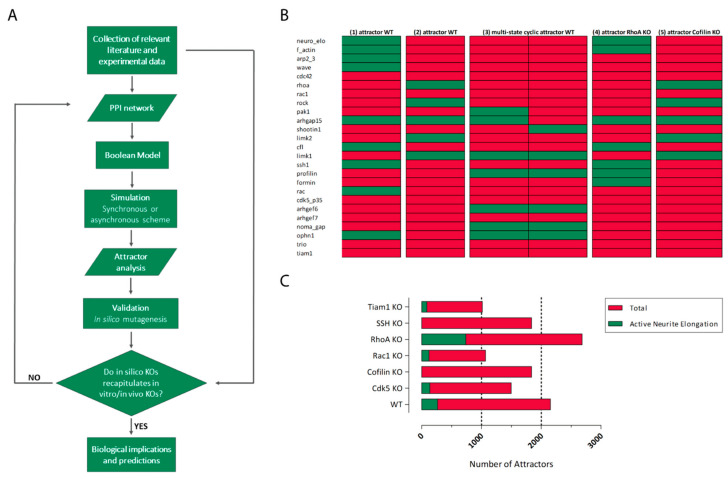
**Boolean network.** (**A**). Flow chart of the procedure to follow in order to construct a Boolean model. (**B**). Attractor analysis of the Boolean network for the study of neurite extension (Table 1). Comparison of five representative attractor profiles: (1) fixed-point attractor of the WT network (active phenotypic node); (2) fixed-point attractor of the WT network (inactive phenotypic node); (3) multi-state cyclic attractor of the WT network (inactive phenotypic node); (4) fixed-point attractor of the RHOA-KO network (active phenotypic node); (5) fixed-point attractor of the Cofilin-KO network (inactive phenotypic node). Red boxes correspond to inactive nodes and green boxes to active nodes. (**C**). In silico mutagenesis for the validation of the Boolean network (Table 1) using published experimental data from KO models (Table 2). Red bars refer to the total number of attractors obtained. The green part of each bar corresponds to the number of attractors with an active phenotypic node computed over all attractors obtained in each simulation.

The simulation aims to identify the steady states reached by the system, named “attractors”, corresponding to particularly stable configurations of activity for each node of the network (Figure 4B). Attractors are thought to correspond to cell phenotypes [367]; consequently, it is a good practice to add in the network one or more “abstract” nodes whose meaning refers to a particular phenotype, like the one named “neurite extension” in Figure 2A, facilitating the identification of attractors that correspond to the activation (or inactivation) of the phenotypic node.

The simulation performed on the neurite elongation Boolean network using the exhaustive method and the synchronous type yielded 2155 attractors (five relevant examples are shown in Figure 4B) in which the phenotypic node is active in 12.4% of attractors (Figure 3C). This preliminary simulation suggests that the system has a low propensity to promote neurite elongation in its default condition. As a validation, we performed an in silico mutagenesis analysis using published experimental data on KO/KD models for all the proteins of the network (Table 2).

The in silico KO is achieved by fixing the state of a particular node to 0 throughout the simulation. The results of these analyses show that, in all cases, the effects of KO observed in real models were confirmed in silico (Figure 4C).

Despite the apparent oversimplification of the Boolean models, they have been successfully used to analyze the dynamics of various biological networks, especially in reverse engineering of regulatory networks and in analyzing complex mutant expression data [62,381,382,383], improving the design of bench experiments.

## 6. Therapeutic Opportunities for Cytoskeleton-Related Forms of ID and Related Conditions

Cognitive deficits in ID are the results of alterations in many cellular processes including neurogenesis, migration, and, consequently, neuronal connectivity. This pathology has its onset during brain development, as high-confidence ID genes are preferentially expressed from early fetal to late mid-fetal stages [14]. Although their expression has a big impact during neuronal development, most of them continue to be expressed during adulthood (e.g., the ones mutated in cytoskeleton-related forms of ID, Figure 2B,C), thus suggesting that they may continue to contribute to the pathological phenotype.

For many years, ID and the other cognitive disorders were thought to be irreversible, especially in adults, and treatments were focused only on personalized educational plans and co-morbidity alleviation, except for a few syndromic forms of ID, for which specific treatments, like enzyme replacement therapy, are used and result in intellectual improvements [384]. Nonetheless, recent studies on specific forms of ID and other NDDs provide evidence that neurological impairments can be reversed, even in postnatal life, thanks to the retained ability of local neuronal circuitries to undergo plastic reorganization and balance excitatory vs. inhibitory synaptic density. Notable examples of targeted treatments on models of NDDs are provided:

(1) Starting from the hypothesis that learning deficits in NF1 (neurofibromatosis 1) are caused by an excess of RAS activity and by the consequent increase in GABA-mediated inhibition, *Nf1*^+/−^ mice (a model of NF1) were treated with both farnesyl-transferase and HMG-CoA reductase inhibitors to decrease RAS activity; the treatment was successful in rescuing spatial memory and attention deficits [385,386].
(2) The observation that the hippocampal signaling through postsynaptic GABA receptors was significantly increased in Ts65Dn mice (a model of DS) prompted the testing of selective GABA_B_ and GABA_A_ receptor antagonists; both treatments rescued memory in novel place and object recognition tests and contextual fear conditioning tasks [387,388,389]. It was later shown that GABA_A_ receptor signaling is excitatory rather than inhibitory in Ts65Dn mice and DS patients, because of an increased hippocampal expression of the cation chloride cotransporter SLC12A2 (solute carrier family 12 member 2). Its inhibitor, bumetanide, a common diuretic, was able to restore synaptic plasticity and hippocampus-dependent memory in adult Ts65Dn mice [390]. Recently, the discovery that the over-activation of microglia plays a role in the DS phenotype widened our knowledge about this pathology, as it has resulted in successful testing of anti-inflammatory drugs to rescue cognitive impairments [391].
(3) As the mutation in *CREBBP* (CREB-binding protein) is considered the most significant mutation in Rubinstein–Taybi syndrome, pharmacological strategies to enhance CREBBP-dependent gene expression were investigated. *Crebbp*^+/−^ mice (a model of Rubinstein–Taybi syndrome) treated with either PDE4 inhibitor (to enhance cAMP signaling) or HDAC inhibitor (to halt the counterpart of the histone acetylation function of CREBBP) were rescued for long-term memory deficit [392,393]. Similarly, *Kmt2d^+/βGeo^* mice (a model of Kabuki syndrome) were rescued by the treatment with HDAC inhibitors [394].
(4) Hyperactivity of MTOR signaling is observed in several neurodevelopmental disorders, the so-called “mTORopathies”; therefore, it is not surprising that MTOR inhibitors were extensively tested. Heterozygous mutations in either *TSC1* or *TSC2* that form an MTOR-inhibiting complex can cause tuberous sclerosis by hyperactivating MTOR signaling. *Tsc2^+/−^* and *Tsc1* homozygous mutant mice (a model of tuberous sclerosis), were treated with rapamycin, rescuing spatial learning and context discrimination deficits together with neurological findings [395,396]. Rapamycin prevented seizures and rescued defective cortical lamination and heterotopia in *Strada*-KO model, an upstream inhibitor of MTORC1, in a rare NDD called Pretzel syndrome [397]. Interestingly, MTOR inhibitors are currently in clinical trials as antiepileptic agents [398]. This class of drugs was also tested to revert neuronal hypertrophy caused by PTEN deficiency in Lhermitte–Duclose and Cowden syndromes [27] and is seen as a promising approach for the treatment of ASD [399].
(5) *Fmr1^−^*^/*−*^ mice (a model of FXS) were used to study the GABA_A_ergic deficit that underlies FXS; treatment with a mGluR5 antagonist rescued associative learning [400], as well as treatments with positive allosteric modulators of GABA_A_ receptors in animal models [401] and GABA_B_ receptor agonists, which, in patients, seemed to rescue behavioral functions [20]. Additionally, the antibiotic minocycline, a metalloproteinase inhibitor, appeared to be effective in patients [402].

Although ID genetic heterogeneity sets a limit on individual treatment, these studies have the merit to demonstrate that ID is potentially responsive to therapeutic interventions. The identification of common disrupted molecular and cellular mechanisms will help in finding flexible therapies, targeting central nodes more than individual genes. One of these nodes is represented by the cytoskeleton under the control of Rho GTPases signaling.

### 6.1. Pharmacological Stabilization of Microtubules

Several genes associated with ID code for proteins acting on microtubules’ formation and regulation [288]. Likewise, alterations of the microtubule cytoskeleton have been linked to ASD, schizophrenia, DS, and major depression disorders [403,404].

Microtubule stabilizers were mostly studied in neurodegenerative disorders characterized by compromised MAPT (microtubule-associated protein tau, or simply tau) functions. According to the hypothesis that microtubule-stabilizing drugs could offset the loss of normal tau functions [405], taxane microtubule stabilizers were tested. Paclitaxel was tested on a mouse model presenting tau pathology in spinal motor neurons [406], restoring fast axonal transport and ameliorating motor impairments. Another taxol, Epothilone D, able to cross the blood–brain barrier, was tested in mouse models of both tauopathy and schizophrenia [407,408], reducing axonal dysfunction, cognitive deficits, and synaptic transmission. Indeed, although taxanes are well-known chemotherapy medications [409], at nanomolar concentrations they appeared safe, eliciting only beneficial effects [407].

Microtubule stabilization can be achieved also by targeting microtubule-binding proteins. The neurosteroid pregnenolone (PREG) was shown to induce CLIP1 active conformation and to restore its association with microtubules in *Cdkl5*-deficient neurons rescuing morphological defects [313].

Another solution was provided by small peptides able to act out the activity of microtubule-associated proteins. NAP (NAPVSIPQ), alias Davunetide, is a neuroprotective peptide snipped by ADNP (activity-dependent neuroprotector homeobox). *ADNP* haploinsufficiency results in increased tau phosphorylation and memory impairments in neurodegenerative diseases, including Alzheimer’s disease [410]. *ADNP* mutations have been linked also to ASD and ID [411]. In preclinical studies, NAP was found to reduce tau hyperphosphorylation and to interact with MAPRE1-3, enhancing microtubule assembly [412]. Interestingly, Risperidone, an atypical antipsychotic, interacts with MAPRE1-3, competing with NAP. Risperidone treatment in schizophrenia and ASD patients improved their cognitive functions and mitigated their disruptive behavior [413,414].

Several other compounds are under investigation for their activity over microtubules and microtubule-related proteins, e.g., blood–brain barrier-penetrant heterocyclic molecules able to stabilize microtubules, and calpain inhibitors, which, by protecting the protein LIS1 (platelet-activating factor acetylhydrolase 1b regulatory subunit 1) against proteolysis, are able to recover retrograde transport and network formation in *LIS1^+/-^* mice (a model of lissencephaly) [415,416].

### 6.2. Pharmacological Modulation of Actin Dynamics

Altered actin polymerization kinetics features most of the ID models presenting mutations on genes involved in actin-reorganizing signaling pathways. Yet, the phenomenon was characterized only in ASD and schizophrenia patients, in which a defective actin polymerization was observed [52,56].

Because of its ubiquitous expression, directly targeting actin raises many concerns. Conversely, a more appealing possibility is ABPs’ or upstream regulators’ targeting, which are largely brain-specific, e.g., CTTNBP2 (cortactin binding protein 2) [417], particularly enriched in specific brain areas, e.g., PAK1 in the prefrontal cortex (Figure 2B) or KALRN in the cortex and hippocampus [418], or specific to neuronal compartments, e.g., KLHL17 (kelch-like family member 17, also known as actinfillin) at the PSD [419].

The validity of such an approach was demonstrated in a study on a *Shank3*-deficient mouse model [280]. First, this study correlated the NMDA receptor hypofunction to an impaired actin polymerization caused by the increased level of active ADF/cofilin, which was in turn caused by the decreased RAC1/PAK1/LIMK1 signaling. Then, it showed how the inhibition of ADF/cofilin through a competitor peptide rescued ASD-like behaviors, improving the F-actin/G-actin ratio and restoring NMDA receptor function in mice.

Actin dissociation/association rate can also be affected by the presence of other natural actin ligands, fungal and bacterial toxins, and cytotoxic macrolides derived from marine sponges. As for ABPs, actin ligands are classified according to their activities and roughly divided into actin stabilizers, e.g., phalloidin, jasplakinolide, and miurenamide A, and destabilizers, e.g., latrunculin, cytochalasin D, and kabiramide C. Both stabilizing and destabilizing agents are intended in their proper meaning only in vitro, while in vivo they are expected to affect the cytoskeleton dynamics only by delaying or accelerating them. These compounds have been largely used as a cell biology tool, like phalloidin in conjugation with fluorophores, overlooking their ability to compete with specific ABPs in a biomimetic mode. Trisoxazole macrolides, e.g., kabiramide C, are small biomimetic molecules promoting actin filament severing and capping by competing specifically with Gelsolin [420]. Analogously, the myxobacterial compound miuraenamide A binds to and stabilizes F-actin by competing with ADF/cofilin for its binding site [421]. Interestingly, low-dose treatment of SKOV3 and HUVECs cells with miuraenamide A showed no effects on cell viability and proliferation, while actin structure was initially subtly changed, but recovered subsequently [422].

Since the reorganization of the cell cytoskeleton is an essential process during metastasis formation, this class of molecules has always been seen as a good candidate for anticancer drug development. However, they could be a starting point in the creation of new compounds that do not influence actin polymerization per se but selectively antagonize specific ABPs.

### 6.3. Modulation of Small GTPases Activity

Several forms of ID are caused by mutations that either increase or decrease Rho GTPases’ signaling. However, it is important to note that both hyper- and hypo-activation of these signaling pathways led to similar phenotypes. A good example is provided by the mouse model bearing RAC1 conditional inactivation at the hippocampal level, which resembles the phenotypic abnormalities reported for the FXS model, which in contrast shows a massive activation of RAC1 [10,423]. This comparison stresses the importance of tight control on GTPases functioning and encourages the research of both positive and negative pharmaceutical modulators.

Several compounds were characterized for their inhibitory activity toward RAC1, which is of particular interest in cancer research. Most of them act by interfering with RAC1-GEF PPI, such as NSC23766 and EHop-016 (which interfere with TRIO and TIAM1) [424,425] and AZA1 (which inhibits both CDC42 and RAC1) [426], or by promoting GTP unloading, such as EHT1864 [427]. Peptide-based approaches have also shown early promise [428].

A striking example of such a pharmacological treatment is provided by the *Ophn1*-KO model of ID, which exhibits hyperactive RHOA signaling. In these mice, the administration of the ROCK/PKA inhibitor Fasudil rescued spine density, hippocampal hyperexcitability, ventricular enlargement, and behavioral abnormalities [170,429]. Fasudil was also able to rescue the decreased neurogenic potential and impaired neurite elongation in OPHN1-deficient human iPSCs [171].

In other cases, the opposite approach is required, i.e., positive modulation of the GTPase activity. Hyper-activation of RAC1 through the inoculation of the bacterial toxin CNF1 has been shown to improve the behavioral phenotype of a mouse model of Rett syndrome and to reverse the astrocytic deficits, which were assumed to have an impact on dendritic maturation [430]. CNF1 activates Rho GTPases through a single glutamine deamidation [431,432], allowing the reshape of the actin cytoskeleton and consequently promoting neurotransmission and synaptic plasticity. CNF1 was shown to enhance working memory for object location and discrimination also in WT mice [433,434].

## 7. Concluding Remarks

Current treatments of ID are largely based on psychosocial measures, environmental enrichment, dedicated educational plans, and motor activity, while pharmacotherapies are lagging. Recent evidence suggests that some phenotypes associated with cognitive disabilities can be reversed, through either genetic approaches [435] or pharmacotherapy. At present, whether these provide a realistic opportunity for treatment remains to be defined.

One obstacle is represented by the high genetic variability observed in ID. SB approaches and integrative tools are beginning to deconvolute and model the core biological processes responsible for the altered circuitry and synaptic dysfunctions in ID. One of these processes is the regulation of cytoskeleton dynamics, whose hubs are the small GTPases of the Rho class. A relevant outcome of our current knowledge is the possibility to leverage the wealth of experimental and mutational data to derive a computational model, through which we will learn more about the behavior of the system.

Rho class GTPases are promising targets for pharmacological intervention and can be positively or negatively modulated by acting upstream, on the regulatory partners, or downstream, on actin stability. To achieve a brain-specific, GTPase-specific, modest, and controlled remodulation, a full characterization of the PPI between GTPases and their regulatory partners is required. Current knowledge led to the identification of several potentially valid compounds, some of which are well characterized for their biological activity, while others still need a proper characterization.

Future efforts should focus on completing our knowledge about the cytoskeleton core regulatory network, considering that relevant elements might still be missing or have been overlooked. Proteomic analyses focused on the human neuronal cytoskeleton are needed, intending to identify novel druggable elements participating in the GTPase regulatory network or neurodevelopmental processes, i.e., neurite elongation, neuronal migration, and adhesion.

Since most of the current knowledge is based on animal models, an area of strong interest is the generation and validation of cellular models of ID based on human neurons. Such models should recapitulate the cortical and hippocampal endophenotype of human ID and offer the possibility to examine the excitatory/inhibitory balance. In light of these observations, human iPSCs offer a valid model to identify new valuable readouts and to start screening compounds able to alleviate ID [436].

## Figures and Tables

**Table 1:** Boolean model of the GTPases network for neurite elongation.

Targets, Factors (1)	Reference
neuro_elo, f_actin	[68]
f_actin, (profilin | formin | arp2_3 | shootin1) & !cfl	[266,349,350,351,352,353]
arp2_3, wave	[351,354]
wave, (rac1 | cdc42) & !cdk5_p35	[351,355,356]
cdc42, (arhgef6 | arhgef7) & !(noma_gap | ophn1)	[166,176,184,193,357]
rhoa, trio | !(ophn1 | tiam1)	[164,358,359]
rac1, (tiam1 | arhgef6 | arhgef7 | trio) & !(arhgap15 | ophn1)	[164,172,184,193,205,358]
rock, rhoa	[360]
pak1, (rac1 | cdc42 | arhgef6 | arhgef7) & !(arhgap15 | cdk5_p35)	[186,361,362,363,364]
arhgap15, !pak1	[363]
shootin1, pak1	[266]
cdk5_p35, f_actin	[154]
limk2, rock	[360]
cfl, ssh1 & !(limk2 | limk1)	[222,365]
limk1, pak1 | !ssh1	[129,224]
ssh1, f_actin	[222,366]

(1) Targets and factors refer to the components (nodes and edges) indicated in Figure 2A rewritten with the BoolNet R package syntax.

**Table 2:** Proteins involved in the regulation of neurite elongation with the corresponding KO/KD phenotypes.

Protein	Gene Mutation (1)	Phenotype	Reference
Rac1	Forebrain-specific KO	Increased number of primary neurites and secondary branches	[95]
RhoA	KO	Increased axon length (significantly greater actin retrograde flow, fewer actin arcs, and substantially longer F-actin bundles)	[368]
Cdc42	KO	Defective axon formation (disrupted cytoskeletal organization, enlargement of the growth cones, and inhibition of filopodia dynamics)	[97]
WAVE1	KO	No effect on neurite growth	[369]
Cdk5	Dominant-negative	Inhibition of neurite outgrowth	[370]
p35	KD	Inhibition of neurite outgrowth	[370]
Arp2/3	KD	Increased number of irregular, shorter, and broader neurites	[371]
PAK1	Dominant-negative	Decreased number of dendrites	[361]
PAK2	Dominant-negative	No effect on the neurite growth	[372]
PAK3	KD	Increased elongation of neuronal processes	[373]
LIMK2	KD	Reduced number of neurite-bearing cells and the mean neurite length	[222]
LIMK1	KD	Reduced number of neurite-bearing cells and the mean neurite length	[222]
ROCK1	Haploinsufficiency	Increased basal and apical dendritic length and dendritic intersections	[374]
ROCK2	Haploinsufficiency	No effect on the neurite growth	[374]
SSH1/SSH2	KD	Decreased neurite extension	[222]
ArhGEF6	KO	Increased neurite length	[184]
ArhGEF7	Cortex-specific KO	Impaired axon formation	[193]
ArhGAP15	KO	Decreased neurite length and branching	[172,375]
TRIO	Neuron-specific KO	Decreased axon length	[376]
Tiam1	KO	Decreased neurite length	[211]
NOMA-GAP	KO	Decreased dendritic branching	[176]
OPHN1	KO	Decreased dendritic tree complexity, i.e., branching	[377]
Cofilin	KO	Inhibited neurite outgrowth	[378]
Profilin1	KD	Impaired axon elongation	[349]
Profilin1	Mutation of the actin-binding domain	Decreased of neurite length	[265]
Formin	KO	Impaired axon elongation	[379]
Shootin1	KD	Inhibited polarization	[380]

(1) Gene mutations referred to in in vitro and in vivo models.

## Data Availability

SysID database (https://www.sysid.dbmr.unibe.ch/, accessed on 6 June 2021); Online Mendelian Inheritance in Man (OMIM) (https://www.omim.org/, accessed on 6 June 2021); The Human Phenotype Ontology (HPO) (https://hpo.jax.org/app/, accessed on 6 June 2021); Gene Ontology Resource (GO) (http://geneontology.org/, accessed on 6 June 2021); Human Brain Transcriptome (HBT) (https://hbatlas.org/, accessed on 6 June 2021); non-neuronal cell types RNA-seq data are available in the NCBI GEO database as GEO: GSE73721.

## Abbreviations

ID	Intellectual Disability
SB	Systems Biology
NDD	Neurodevelopmental Disorder
XLID	X-linked Intellectual Disability
PPI	Protein::Protein Interaction
GO	Gene Ontology
GDD	Global Developmental Delay
ASD	Autism Spectrum Disorder
LOF	Loss Of Function
FXS	Fragile X Syndrome
LTP	Long Term Potentiation
LTD	Long Term Depression,
DS	Down syndrome
G-actin	Glomerular actin
F-actin	Fibrous actin
GAP	GTPase Activating Protein
GEF	Guanine Exchange Factor
KD	Knock-Down
ABP	Actin Binding Protein
NPF	Nucleation Promoting Factor
WRC	WAVE Regulatory Complex
PSD	Postsynaptic Density
+TIP	Microtubule Plus End Tracking Protein
CLIP	Cytoplasmic Linker Protein
